# Joint Passive Detection and Tracking of Underwater Acoustic Target by Beamforming-Based Bernoulli Filter with Multiple Arrays

**DOI:** 10.3390/s18114022

**Published:** 2018-11-18

**Authors:** Zhongyue Chen, Wen Xu

**Affiliations:** 1 College of Information Science and Electronic Engineering, Zhejiang University, Hangzhou 310027, China; joywithjoy@zju.edu.cn; 2 Key Laboratory of Ocean Observation-Imaging Testbed of Zhejiang Province, Zhoushan 316021, China

**Keywords:** Bernoulli filter, target tracking, multiple array processing, beamforming, random finite set

## Abstract

In this paper, improved Bernoulli filtering methods are developed to deal with the problem of joint passive detection and tracking of an underwater acoustic target with multiple arrays. Three different likelihood calculation methods based on local beamforming results are proposed for the Bernoulli filter updating. Firstly, multiple peaks, including both mainlobe and sidelobe peaks, are selected to form the direction-of-arrival (DOA) measurement set, and then the Bernoulli filter is used to extract the target track. Secondly, to make full use of the informations in the beamforming output, not only the DOAs but also their intensities, the beam powers are used as the input measurement sets of the filter, and an approach based on Pearson correlation coefficient (PCC) is developed for distinguishing between signal and noise. Lastly, a hybrid method of the former two is proposed in the case of fewer then three arrays. The tracking performances of the three methods are compared in simulations and experiment. The simulations with three distributed arrays show that, compared with the DOA-based method, the beam-based method and the hybrid method can both improve the target tracking accuracy. The processing results of the shallow water experimental data collected by two arrays show that the hybrid method can achieve a better tracking performance.

## 1. Introduction

In many target detection and tracking applications, detection and estimation are usually carried out separately under the Bayes framework [1]. Hypothesis tests based on the Neyman–Pearson or Bayes criterion [2] are applied to detect the existence of the target. Sequential probability ratio test (SPRT) which makes full use of all collected data based on the continuity of the target state [3] is also a detector. The estimators start working after the target has been declared to be detected. Examples of this type of sequential estimators include the Kalman filter (KF) [4], the extended Kalman filter (EKF) [5], the unscented Kalman filter (UKF) [6], and the particle filter (PF) [7]. Especially, the PF is a sequential Monte Carlo (SMC) method that uses a random particle system (states and weights) to approximate the relevant probability distribution. PF provides a generalized approximate solution for non-linear, non-Gaussian, and unconventional measurement problems where analytical solutions are difficult to obtain [8]. In PF applications, importance sampling is critical. To better apply the particle filter, researchers proposed appropriate resampling methods to reduce the degeneracy of the particle system [9,10]. Gaussian sum particle filter is a special kind of particle filter easier to implement where the relevant probability distribution is assumed as the sum of Gaussian distributions [11]. For low signal-to-noise (SNR) scenarios, track-before-detect (TBD) algorithms arise. In the TBD algorithm, detection is not declared for every frame. Instead, detection is declared when the estimated track is returned after processing of several data frames [12]. In the multi-target tracking problems, multiple hypothesis on measure-to-track association can be filtered to achieve the tracking result. A comparison between multiple hypothesis Kalman filter and the multi-target particle filter can be found in Reference [13].

In passive acoustic target surveillance applications, however, a non-cooperative target may not always radiate a signal that is observable for detection and tracking. The noisy environment increases the uncertainty of obtaining the exact target existence information from available observations. A filter that can distinguish the existence of a target by noisy observations and can be able to resume track after losing the target for a while is needed.

Recently, target tracking based on random finite set (RFS) theory provides new opportunities for state-space processing which has been widely used in many tracking examples, such as video image recognition [14], infrared target tracking [15], and vehicle tracking detection [16]. Mahler and Vo systematically extended RFS theory to target tracking and made great progress in both theoretical research and applications [17]. Bernoulli filter is one of the RFS target tracking methods where the target existence and location state is assumed to be a Bernoulli RFS. It can be used to handle a variety of problems such as multiple on/off switching systems, detection imperfections, and inaccurate estimates [18]. Target tracking with bearings-only measurements by RFS-based filters has been studied from various aspects [19,20,21,22]. In Reference [23], Bernoulli filter tracking along with fusion is applied in an active multi-static acoustic sensor network. Gune considered the problem of detecting and tracking a high-frequency acoustic target in the frequency-azimuth plane obtained by an acoustic vector using Bernoulli filter [24]. However, to the best of our knowledge, modeling and filtering were conducted directly on the direction-of-arrival (DOA) estimations, and the DOA estimation process was usually neglected in the previous research.

In active radar monitoring, the measurement set of Bernoulli filter is assumed to be composed of a DOA estimation of the true target, whose error is a zero mean Gaussian variable, and several DOAs generated by clutters, which form a Poisson RFS [25]. However, the physical basis is different in the study of passive detection and tracking of an underwater target. There are no clutters, but, in estimating target DOA by passive beamforming in a noisy environment, outputs at the sidelobes may be higher than that at the mainlobe corresponding to the target position, resulting in significant errors. To deal with this problem, we jointly use beamforming and Bernoulli filters. A more general assumption is made for the underwater acoustic signal [26], where Gaussian noise is added directly to the received sound pressure. We conduct conventional beamforming (CBF) on the target signal band to process frequency snapshots at distributed arrays separately, and then employ the Bernoulli filter to track the target.

The main contribution of our work is that three different likelihood calculation methods based on local beamforming results are proposed for the Bernoulli filter. Firstly, we construct a DOA measurement set from the passive beamforming results (the DOA-based method). Similar to the method used in Reference [27], to increase the probability of selecting the target DOA, we select the angles corresponding to multiple peaks of the beamforming results as the measurement set. The set contains the DOA estimation corresponding to the real target (mainlobe) and several spurious peaks caused by sidelobes. Secondly, different from the method where only DOA peaks are conserved, we propose to use the beam power as the measurement set instead, because the beam power has not only DOA peak position information but also peak intensity information. Hence, we can use beam pattern to refine the target position (or DOA, or azimuth) from the data beam power. However, the tracking results can be trapped in the end-fire direction of one array where the beam pattern is flat and ambiguous. Therefore, at last, we propose a hybrid method, aiming to combine the advantages of the DOA-based method and the beam-based method in different situations. We use the practical acoustic model to generate sound pressures in the simulations to compare the proposed methods. Filters are also used to process experimental data collected in the real world.

Throughout this paper, we use uppercase calligraphic letters to denote the RFS variable, and ∅ denotes the emptyset. For any RFS S, the cardinality of S is denoted by |S|. Symbol ∖ denotes the set-difference operation. Bold lowercase letters are used to denote column vectors, and bold uppercase letters denote matrices. Spaces of variables are denoted by uppercase blackboard bold letters, where $N_{0}$ is the space of non-negative integers. We use blkdiag(·) to denote block diagonal matrix, where matrices in the brackets are diagonal blocks. $N(m_{x},C_{x})$ is denoted as a Gaussian distribution with mean $m_{x}$ and covariance matrix $C_{x}$, and the corresponding probability density function (PDF) with respect to x is denoted as $N(x;m_{x},C_{x})$. We use *f* as a generic symbol for the finite set statistics (FISST) PDF of the RFS variables, while *p* is denoted as a generic symbol for the traditional PDF of the vector variables. For any variable or function ζ iterated in the prediction and update process of the Bayesian filtering framework, $\zeta_{k|k-1}$ is used to represent the prediction of ζ from time k−1 to time *k*, and $\zeta_{k|k}$ represents the update of ζ with all previous states and measurements from the beginning to time *k*.

The rest of the paper is organized as follows. The Bernoulli filter are briefly reviewed in Section 2, and the prediction step is provided. In Section 3, we establish two kinds of measurement sets derived from beamforming in a multiple-array system, which leads to different update process refers to DOA-based, beam-based, and hybrid Bernoulli filter, correspondingly. Section 4 presents the numerical implementation of the filters for practical usage. The proposed filters are implemented to process data from both simulations and real experiments. The performance results are shown in Section 5, and the paper is finally concluded in Section 6.

## 2. Bernoulli RFS Target State Model and Prediction Process

The concept of RFS was introduced by Mahler to describe the state of a randomly present target or multiple targets [17]. RFS-based Bayes filter aims to solve problems such as detection, estimation, and data association of target tracking within a unified paradigm [17,28].

In the RFS-based Bayes filter, target states and sensor measurements are modeled as RFSs instead of traditional vector formulation. Suppose that $n_{k}$ targets present in the surveillance area at time *k*, and their state vectors are $x_{k,1},\ldots ,x_{k,n_{k}}$, respectively. The state of all the targets at time *k* is presented by an RFS on the state vector space, $X_{k}=\left\{x_{k,1},\ldots ,x_{k,n_{k}}\right\}\in F\left(X\right)$, where X is the state vector space. All the important information about the target, including the target number ($|X_{k}|=n_{k}$) and the dynamic states of each target, has been contained. Likewise, the RFSs of sensor measurements are defined as $Z_{k}=\left\{z_{k,1},\ldots ,z_{k,m_{k}}\right\}\in F\left(Z\right)$, where Z is the measurement vector space.

The posterior probability density of the state variable is propagated in the predicting and updating procedure of the Bayes filter. By using the RFS state and measurement sets, we can extend the traditional Bayes filter from vector space to RFS framework. The predicting and updating process of the RFS posterior densities $f_{k|k}\left(X_{k}|Z_{1:k}\right)$ can be expressed as follows [18]:

(1) $$f_{k|k-1}\left(X_{k}|Z_{1:k-1}\right)=\int \phi_{k|k-1}\left(X_{k}|X_{k-1}\right)f_{k-1|k-1}\left(X_{k-1}|Z_{1:k-1}\right)\delta X_{k-1},$$

(2) $$f_{k|k}\left(X_{k}|Z_{1:k}\right)=\frac{\varphi_{k}\left(Z_{k}|X_{k}\right)f_{k|k-1}\left(X_{k}|Z_{1:k-1}\right)}{\int \varphi_{k}\left(Z_{k}|X_{k}\right)f_{k|k-1}\left(X_{k}|Z_{1:k-1}\right)\delta X_{k}}\;,$$

where $Z_{1:k}=Z_{1},\ldots ,Z_{k}$ is the collection of all the previous measurements and $\varphi_{k}\left(Z_{k}|X_{k}\right)$ is the likelihood function of measurement set $Z_{k}$, given the state $X_{k}$. The RFS state is assumed to be Markovian with transitional density $\phi_{k|k-1}\left(X_{k}|X_{k-1}\right)$. With the updated posterior densities $f_{k|k}\left(X_{k}|Z_{1:k}\right)$ at every time step *k*, RFS state $X_{k}$ can be estimated by criteria such as maximum a posteriori probability (MAP). Although the set integrals in the RFS iteration are usually intractable, solutions can be found for joint detection and tracking of a single target.

For the case of tracking a target which may randomly be present or disappeared in a surveillance area, Bernoulli RFS can be adopted to model the target state, whose cardinality distribution is Bernoulli. Such RFS is either an empty set (with probability 1−q) or a singleton (with probability *q*), whose element x is the state vector of the target distributed following the PDF p(x). Thus, the FISST PDF of a Bernoulli RFS is completely determined by the target existence probability *q* as

(3) $$f\left(X\right)=\left\{\begin{array}{cc} 1-q, & \mathrm{if}\;X=\emptyset \\ q\cdot p(x), & \mathrm{if}\;X=\{x\}\\ 0, & \mathrm{if}\;|X|\geq 2 \end{array}\right..$$

Apparently, the Bernoulli RFS X can be completely determined by the corresponding existence probability *q* and vector PDF q(x). Therefore, predicting and updating the RFS posterior densities $f_{k|k}\left(X_{k}|Z_{1:k}\right)$ are equivalent to predict and update the corresponding existence probability and vector PDF. The predicted existence probability and vector PDF are denoted by $q_{k|k-1}$ and $p_{k|k-1}\left(x\right)$, respectively. $q_{k|k}$ and $p_{k|k}\left(x\right)$ are denoted as the posterior existence probability and vector PDF after update.

The kinetic characterization of the target is used in the prediction stage. This dynamic is described by the Markov transitional density (from time k−1 to *k*):

(4) $$\phi_{k|k-1}\left(X|\emptyset \right)=\left\{\begin{array}{ccc} 1-p_{b}, & \mathrm{if}\; & X=\emptyset \\ p_{b}b_{k|k-1}\left(x\right), & \mathrm{if}\; & X=\{x\}\\ 0, & \mathrm{if}\; & |X|\geq 2 \end{array}\right.,\;\phi_{k|k-1}\left(X|\left\{x^{\prime }\right\}\right)=\left\{\begin{array}{ccc} 1-p_{s}, & \mathrm{if}\; & X=\emptyset \\ p_{s}\pi_{k|k-1}\left(x|x^{\prime }\right), & \mathrm{if}\; & X=\{x\}\\ 0, & \mathrm{if}\; & |X|\geq 2 \end{array}\right.,$$

where $\phi_{k|k-1}\left(X|\emptyset \right)$ describes the birth probability of target with state x, $p_{b}=\mathrm{Pr}(|X|=1||X^{\prime }\left|=0\right)$ is the probability of event “target birth”, and $b_{k|k-1}\left(x\right)$ denotes the distribution of the state of the born target on the vector space, which is defined in advance. When prior knowledge of the target is not available, the distribution of the born target is usually assumed to be a uniform distribution over the vector space. $\phi_{k|k-1}\left(X|\left\{x^{\prime }\right\}\right)$ is the survive probability, $p_{s}=\mathrm{Pr}(|X|=1||X^{\prime }\left|=1\right)$ is the probability of “target survive”, and $\pi_{k|k-1}\left(x|x^{\prime }\right)$ is the transitional density on the vector space, which is related to the motion characteristics of the target. A typical target motion model is the constant velocity (CV) model. In a two-dimensional region, the target state vector is expressed by $x_{k}={\left[x_{k},{\dot{x}}_{k},y_{k},{\dot{y}}_{k}\right]}^{T}$, where $\left(x_{k},y_{k}\right)$ is the Cartesian coordinate of the target and $\left({\dot{x}}_{k},{\dot{y}}_{k}\right)$ is the velocity component, correspondingly. The vector state transition equation of CV model is given by:

(5) $$x_{k}=F_{k}x_{k-1}+v_{k},$$

where $F_{k}=\mathrm{blkdiag}\left(G_{k},G_{k}\right)$ is the state transition matrix, and $v_{k}$ is the zero-mean Gaussian process noise with a covariance matrix $Q_{k}=\mathrm{blkdiag}\left(\Xi_{k},\Xi_{k}\right)$. The diagonal blocks are given by

(6) $$G_{k}=\left[\begin{array}{cc} 1 & T_{k}\\ 0 & 1 \end{array}\right],\;\Xi_{k}=\left[\begin{array}{cc} T_{k}^{3}/3 & T_{k}^{2}/2\\ T_{k}^{2}/2 & T_{k} \end{array}\right]\cdot \varpi_{1},$$

where $T_{k}$ is the sampling interval from time k−1 to time *k*, and $\varpi_{1}$ is the potential acceleration disturbance of the moving target. Therefore, the transitional density is Gaussian, $\pi_{k|k-1}\left(x|x^{\prime }\right)=N\left(x;F_{k}x^{\prime },Q_{k}\right)$.

With the definition of the Bernoulli RFS and its Markov transitional density, the prediction of RFS state PDF can be achieved by predicting the target existence probability and vector PDF as follows [18]:

(7) $$q_{k|k-1}=p_{b}\left(1-q_{k-1|k-1}\right)+p_{s}q_{k-1|k-1},$$

(8) $$p_{k|k-1}\left(x\right)=\frac{p_{b}\left(1-q_{k-1|k-1}\right)b_{k|k-1}\left(x\right)}{q_{k|k-1}}+\frac{p_{s}q_{k-1|k-1}\int \pi_{k|k-1}\left(x|x^{\prime }\right)p_{k-1|k-1}\left(x^{\prime }\right)dx^{\prime }}{q_{k|k-1}}.$$

Equations (7) and (8) completely specify the prediction stage of the Bernoulli filter.

In the next section, we present the update stage for the multi-array passive beamforming system. Different kinds of measurement sets are used, which lead to different forms of the update procedure.

## 3. Measurement Model for Multiple Arrays and Update Process

Using a multi-array system for joint passive detection and tracking of the underwater acoustic target, we first perform beamforming on each local array to obtain the relative DOA information of the target to the local array. Subsequently, in the Bernoulli filter, with known array locations, the tracking of the target state (position and velocity in two-dimensional Cartesian coordinates) is achieved using the beamforming results of all the arrays via triangulation.

### 3.1. Passive Local Array Beamforming

For the convenience of practical implementation, CBF is adopted to process the collected data. We divide the signal band of interest into multiple narrow subbands and perform narrow-band CBF on every subband, and then sum up the subband results to obtain the final beam power.

By fast Fourier transform (FFT), the recorded time domain signal of a given *S*-sensor array is converted to frequency-domain snapshots on *M* narrow subbands. The center frequency for the *m*th subband is denoted by $f_{m}$, m=1,…,M. For every subband, *L* snapshots are collected to form one data frame matrix, $R_{m}=\left[r_{m1},\ldots ,r_{mL}\right]$. The *l*th column vector in matrix $R_{m}$ is an S×1 vector. In the plane wave assumption, for signal oriented from a target with azimuth θ, elevation ψ, and range *r*, the propagation delay difference on the array is described by the steering (or replica) vector $a\left(\theta \right)=\frac{1}{\sqrt{S}}{\left[e^{-jk^{T}p_{1}},\ldots ,e^{-jk^{T}p_{S}}\right]}^{T}$, where $p_{s}$, s=1,…,S, are the Cartesian positions of the array elements, and $k=-\frac{2\pi c}{f_{m}}{\left[\sin\left(\psi \right)\cos\left(\theta \right),\sin\left(\psi \right)\sin\left(\theta \right),\cos\left(\psi \right)\right]}^{T}$ is the wave number of the *m*th frequency bin with *c* being the sound speed. In the shallow water environment, depth is negligible compared to the range between target and array. That is, ψ≈π/2, which leads to cos(ψ)≈0 and sin(ψ)≈1. Thus, in the local array beamforming step, we only need to estimate the azimuth (DOA) of the target. Defining the data sample covariance matrix as $C_{m}=\frac{1}{L}R_{m}R_{m}^{H}$, where the superscript H indicates the conjugate transpose, the beam power of narrow band CBF is then $w_{m}^{H}\left(\theta \right)Cw_{m}\left(\theta \right)$. The weight vector of CBF is exactly the driving vector, $w_{m}\left(\theta \right)=a_{m}\left(\theta \right)$. Summation of the beam powers of all the *M* subbands leads to the basic broadband beamforming of the form

(9) $$P\left(\theta \right)=\sum_{m=1}^{M}w_{m}^{H}\left(\theta \right)C_{m}w_{m}\left(\theta \right).$$

When the featured frequencies of the target are included in the summed subbands, the target DOA will be enhanced in the beam power. The estimation of DOA is then given by $\hat{\theta }={\mathrm{arg max}}_{\theta \in (-\pi ,\pi ]}P\left(\theta \right)$.

In some scenarios, for example, when SNR is low or the array sensor spacing does not meet half-wavelength requirement, strong noise at the sidelobes and grating lobes will degrade the performance of DOA estimation. The correct peak still exists in the beam power but is usually lower than the spurious peaks. In other words, the beam pattern corresponding to the correct azimuth is corrupted but remains in the presence of noises and interferences. For the purpose of making full use of the beamforming results, we propose two kinds of derivative measurements of P(θ) in the following context and use them in the update procedure of Bernoulli filter.

### 3.2. Update Process in Multiple Array Systems

Suppose that a system equipped with *N* distributed arrays is deployed in the surveillance region. Every array in the system provides a measurement set at frame *k*. Denote the measurement set of the *n*th array at frame *k* by $Z_{k}^{\left(n\right)}$, n=1,…,N. As the arrays are usually spatially separated, it is reasonable to make the assumption that measurements obtained at different arrays are statistically independent. Thus, the entire likelihood can be expressed as the product of local likelihood functions of individual arrays:

(10) $$\varphi_{k}\left(Z_{k}|X_{k}\right)=\left\{\begin{array}{ccc} \prod_{n=1}^{N}\eta_{*,1}^{\left(n\right)}\left(\left.Z_{k}^{\left(n\right)}\right|x\right), & \mathrm{if}\;X_{k} & =\{x\}\\ \prod_{n=1}^{N}\eta_{*,0}^{\left(n\right)}\left(Z_{k}^{\left(n\right)}\right), & \mathrm{if}\;X_{k} & =\emptyset \end{array}\right.,$$

where $\eta_{*,1}^{\left(n\right)}\left(\cdot \right)$ and $\eta_{*,0}^{\left(n\right)}\left(\cdot \right)$ denote the likelihood of the *n*th array. $\eta_{*,1}^{\left(n\right)}$ characterizes the similarity between measurement sets to a target-oriented measurement with vector state x. On the contrary, $\eta_{*,0}^{\left(n\right)}$ is the likelihood to noise. Symbol ∗ will be replaced by specific methods in the following context. With the definition of the Bernoulli RFS and the likelihood function of the measurement RFS in Equation (10), the update step can be expressed by updating the target existence probability $q_{k|k}$ and the posterior PDF $p_{k|k}\left(x\right)$ on the vector space, respectively [18],

(11) $$q_{k|k}=\frac{q_{k|k-1}\int \ell_{k}\left(Z_{k}|x\right)p_{k|k-1}\left(x\right)dx}{1-q_{k|k-1}+q_{k|k-1}\int \ell_{k}\left(Z_{k}|x\right)p_{k|k-1}\left(x\right)dx},$$

(12) $$p_{k|k}\left(x\right)=\frac{p_{k|k-1}\left(x\right)\prod_{n=1}^{N}\eta_{1}^{\left(n\right)}\left(\left.Z_{k}^{\left(n\right)}\right|x\right)}{\int p_{k|k-1}\left(x\right)\prod_{n=1}^{N}\eta_{1}^{\left(n\right)}\left(\left.Z_{k}^{\left(n\right)}\right|x\right)dx},$$

where

(13) $$\ell_{k}\left(Z_{k}|x\right)=\prod_{n=1}^{N}\frac{\eta_{1}^{\left(n\right)}\left(\left.Z_{k}^{\left(n\right)}\right|x\right)}{\eta_{0}^{\left(n\right)}\left(Z_{k}^{\left(n\right)}\right)}$$

is the measurement likelihood ratio function.

The likelihood functions $\eta_{*,1}^{\left(n\right)}$ and $\eta_{*,0}^{\left(n\right)}$ are important in the update iteration, and will be introduced in the rest part of this section.

### 3.3. DOA-Based Method

In this section, we discuss the construction method of a DOA-based measurement set. In the case of low SNR, the sidelobe outputs may be higher than the mainlobe in the beam power. However, our simulations showed that, if we conserve certain number of peaks, the correct DOA information of the target has a high probability to be retained, which can thus be transferred to the subsequent filter. Therefore, for each local array, we search for λ largest peaks on the beam power curve P(θ). For an individual array, the azimuths corresponding to these peaks compose the measurement set $Z=\{z_{1},\ldots ,z_{\lambda }\}$. The measurement set can be divided into two parts Z=T∪F, where T is the RFS for the true target DOA, and F is the RFS for the spurious peaks. As mentioned before, true set T can be modeled as a Bernoulli RFS. When the true azimuth is not included in the selected peaks, T is an empty set. At each frame, let peak number λ be randomly generated according to the Poisson distribution with the mean of ν, so that the set F of spurious peaks is approximately a Poisson RFS whose PDF is defined as

(14) $$\kappa \left(F\right)=e^{-\nu }\prod_{z\in F}\nu u(z),$$

where u(z) is the density of the false DOA peak z. For the target generated true azimuth set, the conditional PDF is given by

(15) $$f\left(T|\left\{x\right\}\right)=\left\{\begin{array}{ccc} 1-p_{d}\left(x\right), & \mathrm{if}\;T & =\emptyset \\ p_{d}\left(x\right)p\left(z|x\right), & \mathrm{if}\;T & =\{z\} \end{array}\right.,$$

where $p_{d}\left(x\right)$ is the probability of detecting the object in state x; that is, for a given state x, the probability of the corresponding azimuth peak is selected. Moreover, p(z|x) is the conventional likelihood function of true azimuth measurement due to the object x. Similar to other Bernoulli filters, p(z|x) is assumed to follow a Gaussian distribution $N(z;\theta \left(x\right),\sigma_{B}^{2}\left(x\right))$. The mean value θ(x) is the correct DOA of the target x, and the standard deviation $\sigma_{B}\left(x\right)$ equals to half of the 3-dB mainlobe width of the corresponding beam pattern [29].

In an *N*-array system, let $Z^{\left(n\right)}=T^{\left(n\right)}\cup F^{\left(n\right)}$ be the azimuth peak set of the *n*th array. When the target is absent, X=∅ and the measured peak set is exactly the set of spurious peaks. Therefore, the likelihood is given by

(16) $$\eta_{\mathrm{doa},0}^{\left(n\right)}=\kappa \left(Z^{\left(n\right)}\right).$$

When a target is present, the likelihood is given by

(17) $$\begin{array}{cc} \eta_{\mathrm{doa},1}^{\left(n\right)} & =f\left(\emptyset |\left\{x\right\}\right)\cdot \kappa \left(Z^{\left(n\right)}\right)+\sum_{z\in Z^{\left(n\right)}}f\left(\left\{z\right\}|\left\{x\right\}\right)\cdot \kappa \left(Z^{\left(n\right)}\backslash \left\{z\right\}\right)\\ & =\kappa \left(Z^{\left(n\right)}\right)\left[1-p_{d}\left(x\right)+p_{d}\left(x\right)\sum_{z\in Z^{\left(n\right)}}\frac{p(z|x)}{\nu u(z)}\right]. \end{array}$$

The update process of the DOA-based method is completed by substituting the likelihood functions in Equations (16) and (17) into Equations (11)–(13).

### 3.4. Beam-Based Method

In the DOA-based method, we only retain the DOA peak positions from the beamforming outputs for filtering, but the intensity information in the beam power can also be useful. Therefore, we proposed a beam-based method in this section, where the entire beam power for θ∈(−π,π] of the arrays are used as the measurements, that is, $Z_{k}^{\left(n\right)}\equiv P_{k}^{\left(n\right)}\left(\theta \right)$, n=1,…,N, where $P_{k}^{\left(n\right)}\left(\theta \right)$ denotes the beam power of the signal received by the *n*th array at time *k*. According to the definition of likelihood functions, $\eta_{*,1}^{\left(n\right)}$ should take large value when the beam power is close to those oriented from the true direction, while $\eta_{*,0}^{\left(n\right)}$ gets larger when the beam power is as random as the result of the omnidirectional noise. To get a likelihood function of these features, we propose to use the concept of correlation between the data beam power and the beam pattern for some assumed signal arrival direction.

For a fixed array, the element positions in the array are known a priori. Thus, we have known beam pattern (in power) for a given DOA $\theta_{x}$ of some target state x:

(18) $$B\left(\theta ;\theta_{x}\right)=\frac{1}{M}\sum_{m=1}^{M}{∥w_{m}^{H}\left(\theta \right)a_{m}\left(\theta_{x}\right)∥}_{2}^{2},$$

where ${∥\cdot ∥}_{2}$ is to take the $\ell_{2}$-norm of a vector. The beam pattern of the *n*th array is denoted by $B^{\left(n\right)}\left(\theta ;\theta_{x}\right)$. The correlation between the beam pattern and the noisy beam power can tell the likelihood of measurements to target state. In statistics, the Pearson correlation coefficient (PCC) is a measure of the linear correlation between two variables. The PCC between the measured beam power $P_{k}^{\left(n\right)}\left(\theta \right)$ and beam pattern $B^{\left(n\right)}\left(\theta ;\theta_{x}\right)$ is given by

(19) $$\rho_{k}^{\left(n\right)}\left(x\right)=\frac{\sum_{\theta }\left(P_{k}^{\left(n\right)}\left(\theta \right)-{\bar{P}}_{\theta ,k}\right)\left(B^{\left(n\right)}\left(\theta ;\theta_{x}\right)-{\bar{B}}_{\theta }\left(\theta_{x}\right)\right)}{\sqrt{\sum_{\theta }{\left(P_{k}^{\left(n\right)}\left(\theta \right)-{\bar{P}}_{\theta ,k}\right)}^{2}}\sqrt{\sum_{\theta }{\left(B^{\left(n\right)}\left(\theta ;\theta_{x}\right)-{\bar{B}}_{\theta }\left(\theta_{x}\right)\right)}^{2}}}\;,$$

where ${\bar{P}}_{\theta ,k}$ and ${\bar{B}}_{\theta }\left(\theta_{x}\right)$ are the means of $P_{k}^{\left(n\right)}\left(\theta \right)$ and $B^{\left(n\right)}\left(\theta ;\theta_{x}\right)$ with respect to θ, respectively.

The PCC has a value between −1 and +1, where +1 is the perfect positive linear correlation, 0 is the no-linear correlation, and −1 is the perfect negative linear correlation. While the likelihood functions take values in [0,1], mapping rules from PCC to likelihood functions are required. In our applications, when the local beamforming result does not match the beam pattern from the assumed direction, the PCC is negative or close to 0, indicating that there is only noise in this direction, so that the likelihood function to noise should be larger. On contrast, when the local beamforming result completely matches the beam pattern, PCC is equal to 1, indicating that there is a target in the assumed direction. The closer the PCC is to 1, the larger the target likelihood function should be set.

Note that negative PCCs are considered as mismatch here. Readers may be confused because negative correlations are usually considered as strong correlations in applications such as image recognition. That is, if the direction of a measured image are opposite to the original one, the PCC between them equals to −1, but they are still the same object (highly correlated). However, in our application, the PCC between the local beamforming result and the beam pattern being negative means that peaks appear at the DOA positions that should have been valleys, while false peaks appear at where they should not have been existed. This is not a match in our expectation. If the negative PCC was still considered as strong correlation, we would get wrong DOA estimation.

The desired mapping rules should satisfy the requirements listed above. For this purpose, we first define a non-negative PCC $\rho_{k+}^{\left(n\right)}\left(x\right)$, where $\rho_{k+}^{\left(n\right)}\left(x\right)=\rho_{k}^{\left(n\right)}\left(x\right)$ if $\rho_{k}^{\left(n\right)}\left(x\right)>0$ and $\rho_{k+}^{\left(n\right)}\left(x\right)=0$ if $\rho_{k}^{\left(n\right)}\left(x\right)\leq 0$. Then, we generate likelihood functions from $\rho_{k+}^{\left(n\right)}\left(x\right)$ as follows:

(20) $$\eta_{\mathrm{beam},1}^{\left(n\right)}=\frac{1}{\sqrt{2\pi }\sigma_{\rho }}\exp\left\{-\frac{{\left(\rho_{k+}^{\left(n\right)}\left(x\right)-1\right)}^{2}}{2\sigma_{\rho }^{2}}\right\},\;\eta_{\mathrm{beam},0}^{\left(n\right)}=\frac{1}{\sqrt{2\pi }\sigma_{\rho }}\exp\left\{-\frac{{\left(\rho_{k+}^{\left(n\right)}\left(x\right)-0\right)}^{2}}{2\sigma_{\rho }^{2}}\right\},$$

where $\sigma_{\rho }$ is an adjustable probability mapping scale parameter. The update process of the beam-based method is completed by substituting the likelihood functions in Equation (20) into Equations (11)–(13).

### 3.5. Hybrid Method

The beam-based method is beneficial to improve the accuracy when the beamforming result highly matches the beam pattern of the target DOA. However, since there are wider peaks near the end-fire direction in the beam pattern of an individual local array, the tracking results can sometimes be trapped in the end-fire regions. If there are more than three arrays, the effect of such error can be eliminated thanks to the geometric relations between the arrays. In the case of fewer arrays, this problem leads to localization error. Although the DOA-based method may also have ambiguity problems in the end-fire direction, since only one or two DOA peaks are retained for filtering, it is less affected than the beam-based method where the operation of calculating the PCC will aggravate the performance degradation caused by the ambiguity in the end-fire direction. Therefore, the method using DOA measurements only is advantageous for protecting the true value from the effects of the end-fire direction in the filtering procedure, especially when the number of arrays is not enough. Both methods have their own advantages and disadvantages. To complement each other, we propose a hybrid approach combining these two choices. We define the new likelihood functions as the geometric mean of likelihood functions of the DOA-based and beam-based methods as follows:

(21) $$\eta_{\mathrm{hybrid},0}^{\left(n\right)}={\left[\eta_{\mathrm{beam},0}^{\left(n\right)}\right]}^{\alpha }{\left[\eta_{\mathrm{doa},0}^{\left(n\right)}\right]}^{1-\alpha },\;\eta_{\mathrm{hybrid},1}^{\left(n\right)}={\left[\eta_{\mathrm{beam},1}^{\left(n\right)}\right]}^{\alpha }{\left[\eta_{\mathrm{doa},1}^{\left(n\right)}\right]}^{1-\alpha },$$

where α∈[0,1] is the portion coefficient to adjust the weights of beam-based and DOA-based likelihood in the hybrid method. When α=1, the function is the same as the beam-based likelihood, while α=0, it is exactly the same as that of the DOA-based method.

## 4. SMC Implementation of the Bernoulli Filter

The PF/SMC method provides a general framework for the implementation of Bernoulli filter [18]. The PDF $p_{k|k}\left(x\right)$ is approximated by a particle system ${\left\{w_{k}^{\left(i\right)},x_{k}^{\left(i\right)}\right\}}_{i=1}^{N_{p}}$, where $x_{k}^{\left(i\right)}$ is the state of particle *i* and $w_{k}^{\left(i\right)}$ is the normalized particle weight, that is, $\sum_{i=1}^{N_{p}}w_{k}^{\left(i\right)}=1$. The PDF is approximated by

(22) $${\hat{p}}_{k|k}\left(x\right)=\sum_{i=1}^{N_{p}}w_{k}^{\left(i\right)}\delta_{x_{k}^{\left(i\right)}}\left(x\right),$$

where $\delta_{b}\left(\cdot \right)$ is the Dirac function at point b. In the prediction stage, the prediction of PDF in Equation (8) is completed by predicting the particle system

(23) $${\hat{p}}_{k|k-1}\left(x\right)=\sum_{i=1}^{N_{p}+N_{b}}w_{k|k-1}^{\left(i\right)}\delta_{x_{k|k-1}^{\left(i\right)}}\left(x\right).$$

The particles are divided into two parts: $N_{b}$ newly born particles and $N_{p}$ persistent particles. The states of the birth particles are drawn from a pre-defined birth distribution, for example, uniform distribution. The birth particles are equally weighted by $w_{k|k-1}^{\left(i\right)}\propto p_{b}\left(1-q_{k-1|k-1}\right)\cdot \frac{1}{N_{b}},\;i=1,\ldots ,N_{b}$. The persistent particles are the extension of those particles at the previous time. Their states are drawn from the state transitional density $x_{k|k-1}^{\left(i\right)}∼N\left(F_{k-1}x_{k-1}^{\left(i\right)},Q_{k-1}\right)$ for $i=1,\ldots ,N_{p}$. The relationship between the weights of the persistent particles and the previous particle weights is given by $w_{k|k-1}^{\left(i\right)}\propto p_{s}q_{k-1|k-1}\cdot w_{k-1}^{\left(i\right)},\;i=1,\ldots ,N_{p}$.

Note that we use proportion rather than equality, because the weights will also be normalized in the prediction stage.

In the update equations of target existence probability, the integrals can be approximated using predicted particles as

(24) $$\int \ell_{k}\left(Z_{k}|x\right)p_{k|k-1}\left(x\right)dx\approx \sum_{i=1}^{N_{p}+N_{b}}\ell_{k}\left(Z_{k}|x_{k|k-1}^{\left(i\right)}\right)w_{k|k-1}^{\left(i\right)}.$$

To update PDF $p_{k|k}\left(x\right)$, the weights of particles are updated by

(25) $$w_{k|k}^{\left(i\right)}\propto \varphi \left(Z_{k}|\left\{x_{k|k-1}^{\left(i\right)}\right\}\right)w_{k|k-1}^{\left(i\right)}.$$

At the end of the update procedure, sampling importance resampling ($N_{p}$ particles persist) is adopted to regularize the particle system [9]. With all these particles, the MAP estimation of the target state is approximately the weighted summation of the updated particles at time *k*, that is, $\sum_{i}^{N_{p}}w_{k}^{\left(i\right)}x_{k}^{\left(i\right)}$.

## 5. Performance Tests

In this section, performance of the three Bernoulli filters is evaluated using simulational and experimental data.

### 5.1. Simulation Results

**Figure 1:**
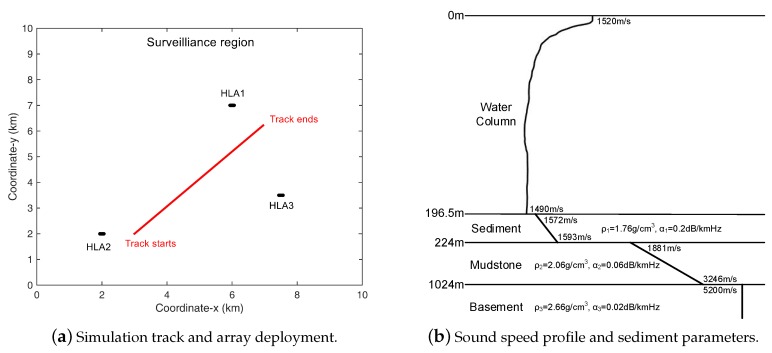
Simulation environment: (**a**) The surveillance region with three HLAs shown by black dots, and the red line is the target track. (**b**) The shallow water environment used in the simulation. The sound speed profile is chosen from the SWellEx96 experiment scenarios. The bottom is modeled as a sediment overlying a mudstone layer. The layer parameters are shown in the figure.

In the simulations, we test the performance of the DOA-based, beam-based and hybrid methods in a shallow water environment, as depicted in Figure 1. As shown in Figure 1a, in a 10 km × 10 km surveillance region, three horizontal line arrays (HLA) of length 256 m each with 24 uniformly spaced hydrophones are deployed at the seabed (of depth about 200 m). The centers of the HLAs are (6.0, 7.0) km (HLA1), (2.0, 2.0) km (HLA2) and (7.5, 3.5) km (HLA3), respectively. The HLAs are drawn as black dots in the figure. An acoustic target at depth 50 m moves with a constant velocity ${\dot{x}}_{t}=1.5$ m/s and ${\dot{y}}_{t}=1.6$ m/s. The source trace used is also presented by the red line. The source we test starts to appear from frame k=10 at (3.0, 2.0) km and finally disappears at frame k=185.

The featured frequency of the target signal is 50, 60, 70 and 80 Hz. The frequency snapshot of the received signal of the *n*th array at time *k* is given by $r_{k}^{\left(n\right)}=A_{k}g_{k}^{\left(n\right)}+n_{k}^{\left(n\right)}$, where $A_{k}$ is the complex Gaussian random amplitude of the source signal following the distribution $\mathrm{CN}(0,\sigma_{s}^{2})$. The Greens’ function vector $g_{k}^{\left(n\right)}$ represents the transmission loss from target location $x_{k}$ to the sensors in array *n*. Additive complex Gaussian noise is denoted by $n_{k}^{\left(n\right)}$ which follows the distribution $\mathrm{CN}(0,\sigma_{n}^{2}I)$, where 0 is a zero mean vector and I is the identity matrix of compatible dimension. Noises at different arrays are assumed to be independent and identically distributed. The source level and noise level are given by $\mathrm{SL}=10\log\sigma_{s}^{2}$ and $\mathrm{NL}=10\log\sigma_{n}^{2}$, respectively. The received data are generated on the frequency band from 40 Hz to 100 Hz with interval Δf=1 Hz. For the featured frequencies of the target, the simulated frequency snapshots are generated containing the signal component, and for simplicity the level of the source signal of each featured frequency is assumed to be identical, i.e., $\sigma_{s}^{2}\left(f_{m}\right)=\sigma_{s}^{2}$. To get data that better fit the realistic shallow water environment, normal mode model KRAKEN is used to generate the transmission loss $g_{k}^{\left(n\right)}$ at signal frequencies for all the arrays with environment parameters shown in Figure 1b. For the other frequency bins in the received bandwidth, noise-only data snapshots are generated. In processing, sliding window is used to pick the snapshots. One single frame *k* with L=11 snapshots are used for beamforming at one time with five snapshots overlapping between the adjacent frames. The sample time interval between snapshots are set to be 2.5 s and thus the time interval in between frames equals to 15 s.

There are two kinds of criteria here to evaluate the filter performance. Optimal subpattern assignment (OSPA) is a measure that is used to analyze the estimation error of the target state and target number at the same time. The OSPA distance between two RFS $X=\{x_{1},\ldots ,x_{n_{x}}\}$ and $Y=\{y_{1},\ldots ,y_{n_{y}}\}$, $n_{x},n_{y}\in N_{0}$, is given by

(26) $${\bar{d}}_{p}^{\left(c\right)}\left(X,Y\right)={\left(\frac{1}{n_{y}}\left(\min_{\pi }\sum_{i=1}^{n_{x}}d^{\left(c\right)}{\left(x_{i},y_{\pi (i)}\right)}^{p}+c^{p}\left(n_{y}-n_{x}\right)\right)\right)}^{1/p},$$

if $n_{x}\leq n_{y}$, where $d^{\left(c\right)}\left(x,y\right)=\min\left(c,d\left(x,y\right)\right)$ is the distance between x and y, and d(x,y) is the traditional distance between vectors. If $n_{y}\leq n_{x}$, then ${\bar{d}}_{p}^{\left(c\right)}\left(X,Y\right)={\bar{d}}_{p}^{\left(c\right)}\left(Y,X\right)$. The summation is taken over all permutations π on $\left\{1,2,\ldots ,n_{y}\right\}$. ${\bar{d}}_{p}^{\left(c\right)}$ is the OSPA metric of order *p* with cut-off *c*. Here, we use the Euclidean distance $d\left(x,y\right)={∥x-y∥}_{2}$. The cut-off distance *c* represents the upper bound of the estimation error of the state vector.

Another metric used is to measure the probability of target lost in the tracking problem, called circular position error probability (CPEP), defined as

(27) $$\mathrm{CPEP}\left(r\right)=\frac{1}{\left|X\right|}\sum_{x\in X}\mathrm{Pr}\left\{∥H\hat{x}-Hx∥>r,\forall \hat{x}\in \hat{X}\right\},$$

where matrix H is used to extract components of state vector for performance analysis, and *r* is the pre-defined position error radius, outside which the estimation is regarded as target missing.

Given the target track in Figure 1a, we set NL = 70 dB, and Bernoulli SMC filters are applied for SL =130 dB and SL = 120 dB, respectively. Based on experience, the Bernoulli filter parameters are set to $N_{p}=5000$, $N_{b}=5000$, $p_{s}=0.99$ and $p_{b}=0.01$. When calculating the particle weights for DOA-based method, we set ν=6 and u(z)=1/2π (uniform distribution of azimuth). Probability of detection is assumed to be constant $p_{d}=0.2$. Probability of target existence, CPEP, and OSPA of both location and velocity tracking are compared for the DOA-based, beam-based and hybrid methods. To eliminate the variation caused by SMC implementation, results are averaged over 500 trails with the same filter parameters. Probability of target existence is the updated probability $q_{k|k}$ in the filter iteration. Target is declared when $q_{k|k}>0.5$ and the state vector estimation is accepted. For $q_{k|k}<=0.5$, target is not detected and the RFS state is an empty set. When calculating CPEP, we use H=diag(1,0,1,0) and r=2 km. That is, when the positioning error exceeds 2 km, the target is regarded as lost. At last, we extract the position and velocity components of the state estimate to calculate the OSPA separately so that the location and the speed estimation error can be compared individually. The error cut-off is c=4 km for location and c=0.1 m/s for velocity.

**Figure 2:**
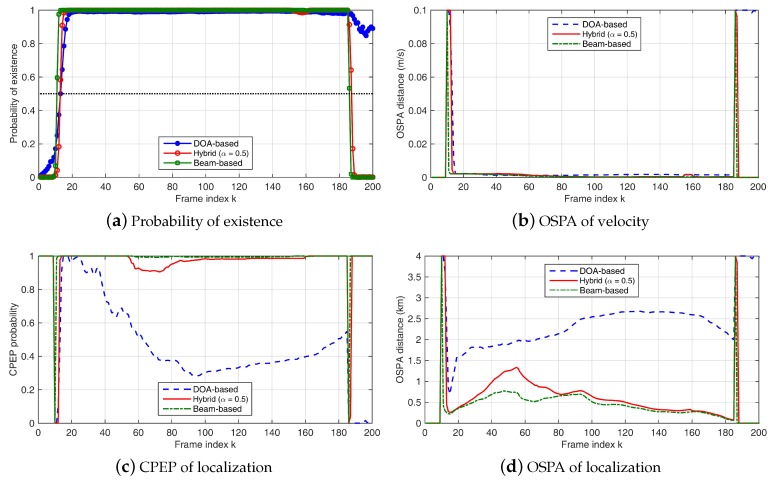
Simulation results with SL = 130 dB and NL = 70 dB: (**a**) probability of existence; (**b**) OSPA error (m/s) of target velocity tracking; (**c**) CPEP of target location tracking; and (**d**) OSPA error (m) of target location tracking. Results of the DOA-based Bernoulli filter are shown in blue lines, results of the beam-based method are presented by green lines, and results of the hybrid method with α = 0.5 are shown in red lines.

**Figure 3:**
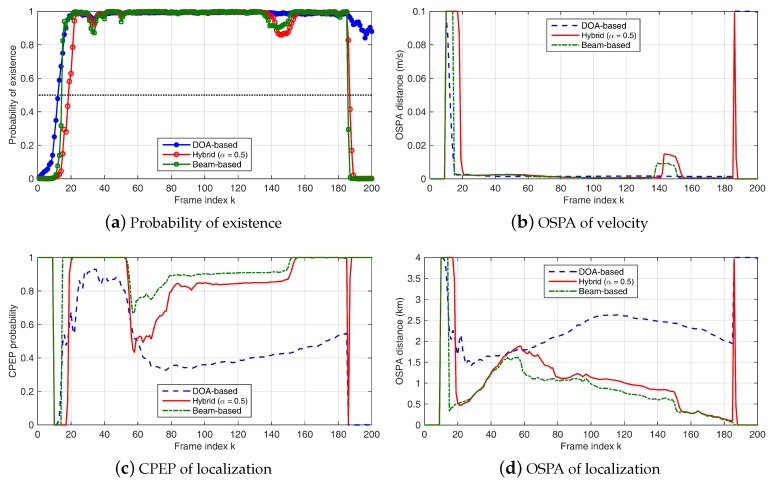
Simulation results when SL = 120 dB and NL = 70 dB: (**a**) probability of existence; (**b**) OSPA error (m/s) of target velocity tracking; (**c**) CPEP of target location tracking; and (**d**) OSPA error (m) of target location tracking. Results of the DOA-based Bernoulli filter are shown in blue lines, results of the beam-based method are presented by green lines, and results of the hybrid method with α = 0.5 are shown in red lines.

Results are shown in Figure 2 and Figure 3. Both beam-based and hybrid methods can better capture the appearance and disappearance of the target signal. The DOA-based method fails to detect the disappearance of the target after k=185. When the target appears, it takes several frames for the beam-based and hybrid filters to detect the target. After the target is detected, the tracking precision of the beam-based and hybrid methods is better than that of the DOA-based method in the simulations. The target track after appearance can be roughly divided into three parts. In the first part, the target is close to HLA2 and is far away from the other two HLAs. As the target moving away from HLA1, the tracking performance degrades (OSPA increases and CPEP decreases). In the middle of the track where target is not close to any of the three arrays, the tracking performance is relatively poor. When the target comes into the area near HLA1 and HLA3, performance improvement can be observed. The performance change is more pronounced when SL = 120 dB. In Figure 3, a possible reason for the sudden change around k=150 is that the target enters a relatively high SNR region of HLA3 (the relationship between performance and SNR is not linear), so the target location is corrected. However, while the target location is corrected, the filter actually detects a large change in the received signal, so that the target existence probability decreases in a short time. The sudden correction of the target position increases the estimation error of target velocity. These errors are corrected after a short period of time. When SL = 130 dB, similar but insignificant performance changes can be observed in the same period. Both beam-based and hybrid methods, where the amplitude information of beamforming is used, are more sensitive to degradation of signal quality. However, in most frames, both beam-based and hybrid methods still outperform the DOA-based method.

### 5.2. Experiment Data Processing Results

**Figure 4:**
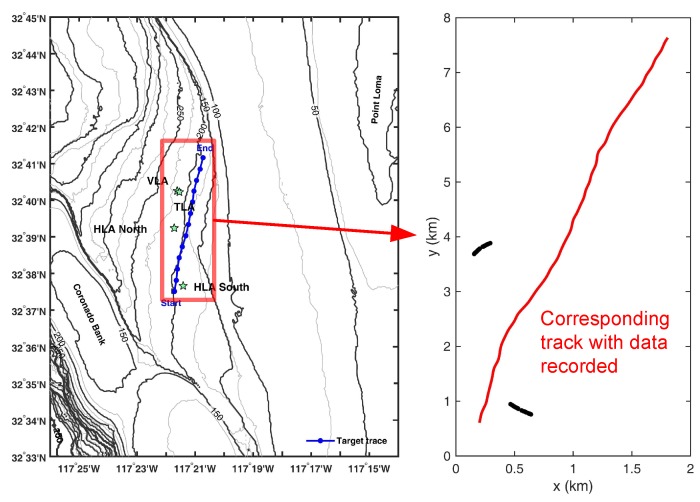
The experiment setup of SWellEx-96 Event S5 is shown in the left figure. The deployed arrays are represented by the stars and the ship-recorded target trace is the blue line. The area in the red rectangle is zoomed in with converted Cartesian coordinates. The data recorded by two HLAs are used for analysis and the HLAs are black lines in the right figure. The red line is the target track corresponding to the data for analysis.

The experiment dataset used to evaluate the performance was collected in the experiment SWellEx-96 Event S5 [30]. In this event, sound pressure was recorded by multiple arrays, a vertical line array (VLA), a tilted line array (TLA) and two HLAs. The experiment area along with the array locations and ship-recorded target trace are shown in Figure 4. The locations of arrays and target trace with recorded data (in the red box) in latitude and longitude are converted to Cartesian coordinates and a surveillance region of size 2 km × 8 km is formed. The corresponding latitude and longitude of the Cartesian origin are $32^{\circ }37.25^{\prime }$ N and $117^{\circ }21.82^{\prime }$ W. For the purpose of tracking the target in the XY-plane, only the data collected by 2 HLAs, referred to as HLA north (HLAn) and HLA south (HLAs), are used. Those arrays are deployed at the seabed with the water depth of 213 m (HLAn) and 198 m (HLAs), respectively. The number of effective array elements for processing is 27 (HLAn) and 28 (HLAs), respectively. Two multi-tone sources travel in the region at speed around 5 knots. The respective source depths are 54 m and 9 m, and their transmitted tone frequencies are listed in Table 1. Data from frequency band of 40–150 Hz are used to test our Bernoulli tracking algorithms. Array element locations as provided on the dataset website are used to calculate the beam pattern in advance.

The recorded sound pressure is sampled at 3276.8 Hz. Data flow is transformed to frequency-domain snapshots with 8192-point FFT. The snapshots length is thus 2.5 s (0.4 Hz frequency bin widths). Similar to the simulations in the previous section, in beamforming, one single frame *k* contains L = 11 snapshots with 5 snapshots overlapping between the adjacent frames. A total of 199 frames were processed. During the experiment, for frames 48∼54, 120∼123 and 131∼134, the deep source stopped projecting continuous wave (CW) tones and started projected chirps that are not on the processed frequency bands. Target is treated as absent during those frames for that only noise is contained in the data for processing.

**Figure 5:**
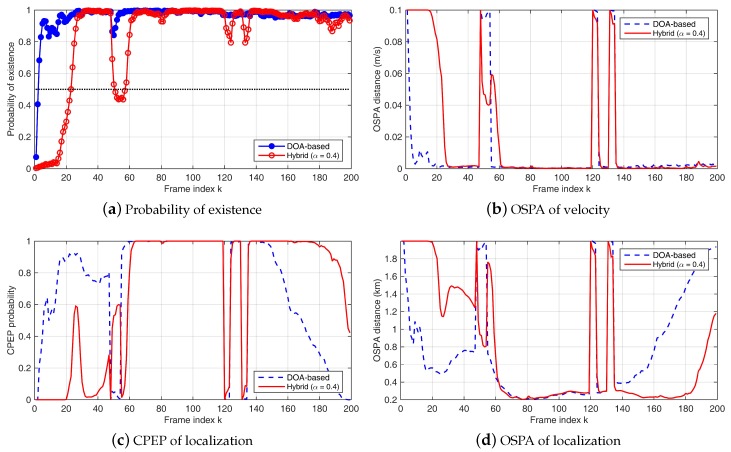
Processing results of data from 2 HLAs in SWellEx-96 Event S5: (**a**) probability of existence; (**b**) OSPA error (m/s) of target velocity tracking; (**c**) CPEP of target location tracking; and (**d**) OSPA error (m) of target location tracking. Results of the DOA-based Bernoulli filter are shown in blue lines, and results of the hybrid method with α = 0.4 are shown in red lines.

In the experiment data processing, the Bernoulli filter parameters are set to $N_{p}=10,000$, $N_{b}=8000$, $p_{s}=0.9$ and $p_{b}=0.1$. In DOA-based method, ν=6 and u(z)=1/2π (uniform distribution of azimuth). Probability of detection is $p_{d}=0.9$. Probability of target existence, CPEP, OSPA of both location and velocity tracking are presented in Figure 5. Results are the average of 500 trails with the same filter parameters. Probability of target existence is the updated probability $q_{k|k}$ in the filter iteration. Target is declared when $q_{k|k}>0.5$ and the state vector estimation is accepted. For $q_{k|k}<=0.5$, target is not detected and the RFS state is an empty set. H=diag(1,0,1,0) and location error radius r=1 km in CPEP metric. Position and velocity OSPA cut-off are set to c=2 km for location and c=0.1 m/s for velocity.

Only two arrays in the experiment led to wrong estimation when the correlation deviated for any of the arrays. This deviation should have been eliminated if we had one more array. Besides, for many frames, the target is in the end-fire direction of HLAs, where the performance of the beam-based method is not satisfactory. In Figure 5, the performance results of the DOA-based method and the hybrid method with α=0.4 are presented. The performance of the beam-based method decreases dramatically, because of the lack of arrays and the effect of the end-fire direction, and is thus not shown. Similar to the simulation results, compared to the DOA-based algorithm, the hybrid method is better at detection, i.e., capturing the appearance and disappearance of the target signal. This can be seen between frames 48∼54, 120∼123 and 131∼134 when the target signal is not included in the processed data. Correspondingly, when using the hybrid method, the convergence rate for the relatively more precise estimation is slower than that of the DOA-based method (for frames 1∼26 at the beginning). From frames 62∼118, both tracking algorithms achieve good performance. Near the end of the track (frames 135∼199), the hybrid method outperforms the DOA-based method.

## 6. Conclusions

We studied the passive joint detection and tracking problem of underwater targets using multiple arrays. The beamforming results obtained by the spatially distributed arrays were used as inputs to the Bernoulli filter to track the target presence, target position, and velocity. Three different likelihood calculation methods, the DOA-based, beam-based and hybrid methods are proposed for the Bernoulli filter. In the DOA-based Bernoulli filter, measurement sets were composed of DOA peak positions of beam power. The beam-based Bernoulli filter adopted the entire beam power as the input measurement set. The hybrid method was proposed to complement the advantages of the DOA-based and the beam-based method in the case of fewer arrays. We used a realistic underwater acoustic waveguide model to generate simulation data and evaluate the performance of the three methods. We also compared their performance using the low-frequency portion of the real data collected in the SWell-Ex96 experiment. The simulation results with three arrays showed that the proposed beam-based method and hybrid method can improve the tracking performance compared with the DOA-based filter. In the experimental data processing with two arrays, results also indicate that the proposed hybrid method has a better ability of detection, as well as a better tracking precision after convergence. It is worth noting that the RFS-based Bernoulli filter has great potential in multi-target tracking. We wish to extend the proposed methods to track multiple acoustic targets in future efforts.

## Figures and Tables

**Table 1:** Frequencies of the Multi-tone sources.

Deep Source (54 m) Frequencies (Hz)	
49 64 79 94 112 130 148 166 201 235 283 338 388	High Signal Level
52 67 82 97 115 133 151 169 204 238 286 341 391	2nd Set of Tonals
55 70 85 100 118 136 154 172 207 241 289 344 394	3rd Set of Tonals
58 73 88 103 121 139 157 175 210 244 292 347 397	4th Set of Tonals
61 76 91 106 124 142 160 178 213 247 295 350 400	5th Set of Tonals
**Shallow Source (54 m) Frequencies (Hz)**	
109 127 145 163 198 232 280 335 385	
